# Supplementary material for: CTP regulates membrane-binding activity of the nucleoid occlusion protein Noc
Source: Mol Cell. 2021 Sep 2;81(17):3623–3636.e6. doi: 10.1016/j.molcel.2021.06.025 (PMC8429893; doi:10.1016/j.molcel.2021.06.025)
Supplement: Document S1. Figures S1–S7 and Tables S1–S3 [file mmc1.pdf]

Molecular Cell, Volume *81*

Supplemental information

CTP regulates membrane-binding activity

# of the nucleoid occlusion protein Noc

Adam S.B. Jalal, Ngat T. Tran, Ling J. Wu, Karunakaran Ramakrishnan, Martin Rejzek, Giulia Gobbato, Clare E.M. Stevenson, David M. Lawson, Jeff Errington, and Tung B.K. Le

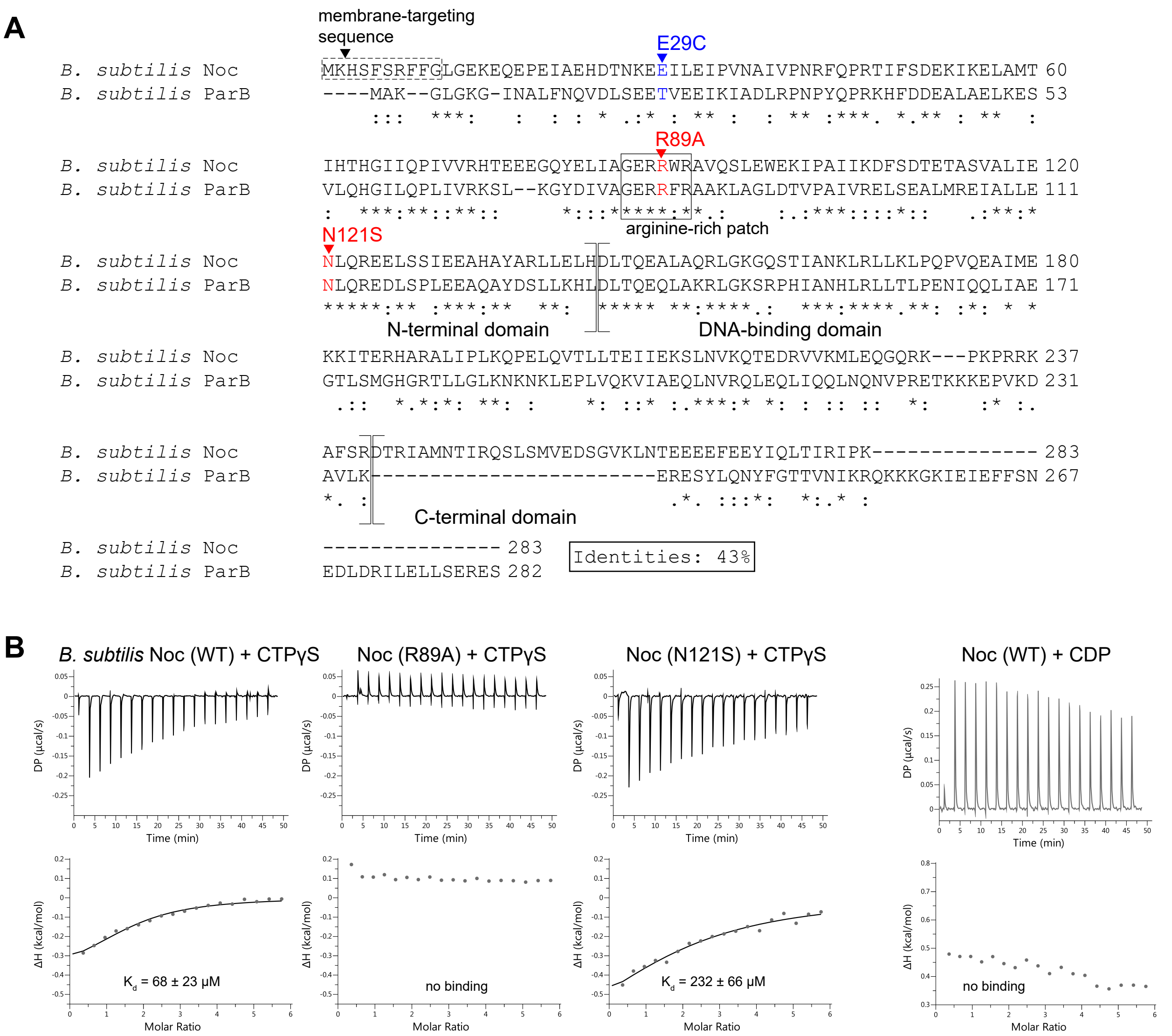

Figure S1. Interactions between Noc (WT and variants) and CTPɣS or CDP, related to Figure 1. (A) A sequence alignment between *B. subtilis* Noc and its paralog ParB. Residues R89 (red) and N121 (red) in *B. subtilis* Noc, whose equivalent substitutions in *B. subtilis* ParB have been shown to impair spreading and CTP binding (Soh et al., 2019), were substituted by alanine and serine, respectively. Residue E29 (blue) was substituted by cysteine to generate a Noc (E29C) variant which was subsequently used in BMOE crosslinking assays (Figure 2A-D). An equivalent substitution T22C was previously used in BMOE crosslinking assays for in *B. subtilis* ParB (Soh et al., 2019). The conserved arginine-rich patch that mediates CTP binding in *B. subtilis* ParB is shown in a solid box. The 10-amino-acid membrane-targeting sequence of Noc is shown in a dashed box. *B. subtilis* ParB does not possess an equivalent membrane-targeting sequence. The positions of the N-terminal domain, the DNA-binding domain, and the C-terminal domain of Noc and ParB are also indicated on the sequence alignment. **(B)** Analysis of the interaction of *B. subtilis* Noc (WT and mutants) with CTPɣS or CDP by isothermal calorimetry (ITC). ITC directly measures the heat released or absorbed during a biomolecular binding event. The large heat exchange from Noc-*NBS* DNA binding (nM binding affinity (Jalal et al., 2020b)) might mask the weaker heat signal from Noc-CTPγS/CDP binding, therefore *NBS* DNA was not included in these ITC experiments. Each experiment was duplicated. Regression curves were fitted, and binding affinities (K_d) are shown.

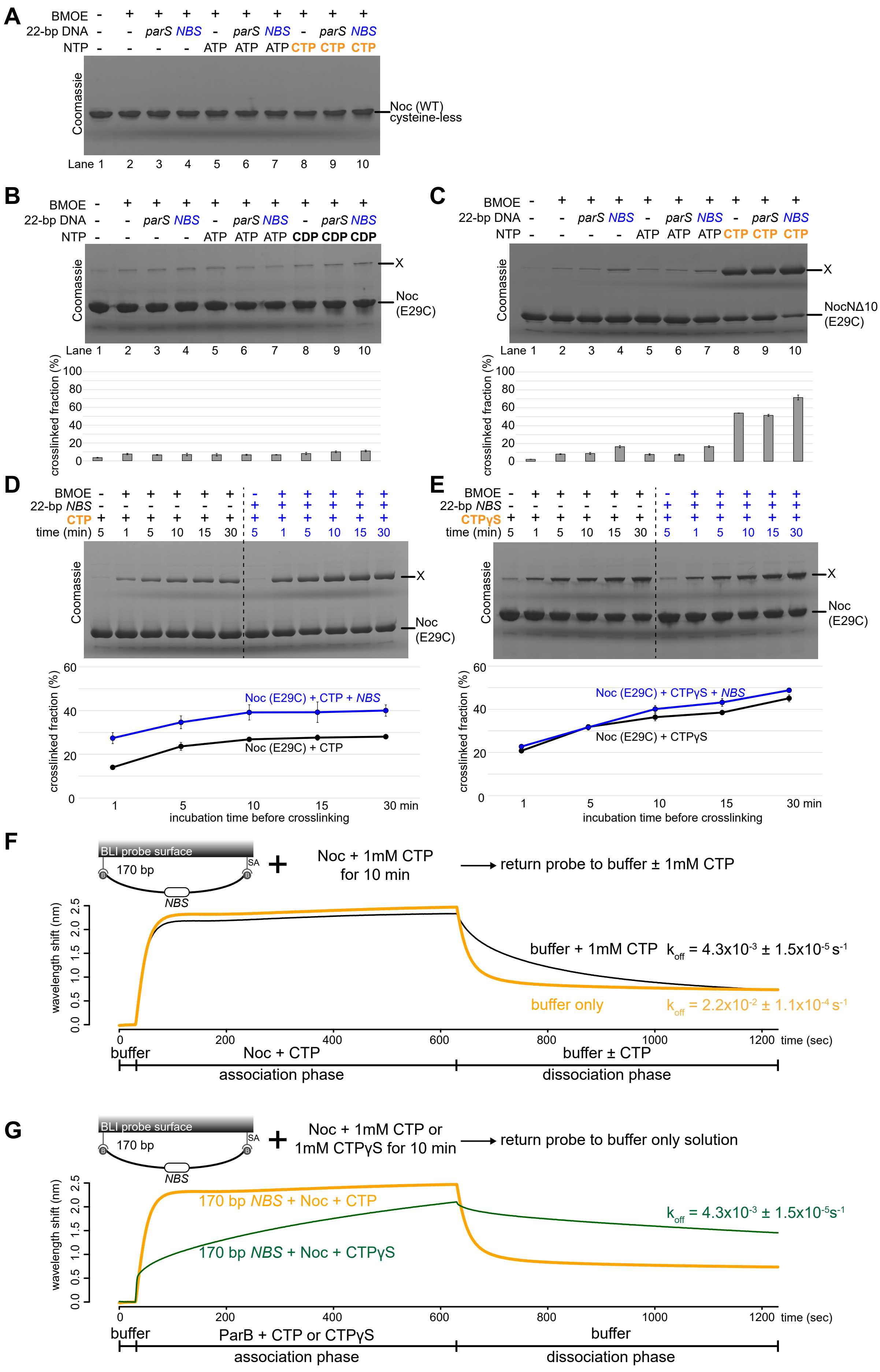

Figure legend (next page)

Figure S2. CTP and CTPɣS, but not other nucleotides, promote the engagement of the N-terminal domain of Noc, related to Figure 2. (A) SDS-PAGE analysis of BMOE crosslinking products of 10 µM *B. subtilis* Noc (WT) protein ± 1.0 µM 22-bp *parS*/*NBS* DNA ± 1.0 mM NTP. Wild-type Noc naturally lacks cysteine, hence does not crosslink in the presence of BMOE. All crosslinking reactions were performed at 22°C unless indicated otherwise. **(B)** Same as panel A but Noc (E29C) was used instead. X indicates a crosslinked form of Noc (E29C). Quantification of the crosslinked fraction is shown below each representative image. Error bars represent SEM from three replicates. **(C)** Same as panel A, but NocNΔ10 (E29C) was used instead. **(D)** Time-course of Noc (E29C) crosslinking with CTP in the presence or absence of 22-bp *NBS* DNA. Purified Noc (E29C) was preincubated with 1.0 mM CTP ± 1 µM 22-bp *NBS* DNA for 1, 5, 10, 15, or 30 min at 4°C (instead of the usual 22°C) before BMOE was added. A lower incubation temperature was needed to slow down the reaction. Quantification of the crosslinked fraction is shown below each representative image. Error bars represent SE from three replicates. **(E)** Same as panel D, but 1.0 mM CTPɣS was used instead. **(F)** BLI analysis of the interaction between *B. subtilis* Noc-CTP and a 170-bp dual biotin-labeled *NBS* DNA. For the association phase, the interaction between a premix of 1.0 µM *B. subtilis* Noc ± 1.0 mM CTP and a 170-bp dual biotin-labeled *NBS* probe was monitored in real-time for 10 min. For the dissociation phase, the probe was returned to either a buffer-only solution or a buffer supplemented with 1mM CTP. The dissociation rate (k_{off}) of bound Noc into buffer is shown for each reaction. **(G)** BLI analysis of the interaction between *B. subtilis* Noc-CTP or Noc-CTPɣS and a 170-bp dual biotin-labeled *NBS* DNA. For the association phase, the interaction between a premix of 1.0 µM *B. subtilis* Noc + 1.0 mM CTP or CTPɣS and a 170-bp dual biotin-labeled *NBS* probe was monitored in real-time for 10 min. For the dissociation phase, the probe was returned to a buffer-only solution. The dissociation rate (k_{off}) of bound Noc into buffer is shown for each reaction. Each experiment was triplicated and a representative sensorgram was shown.

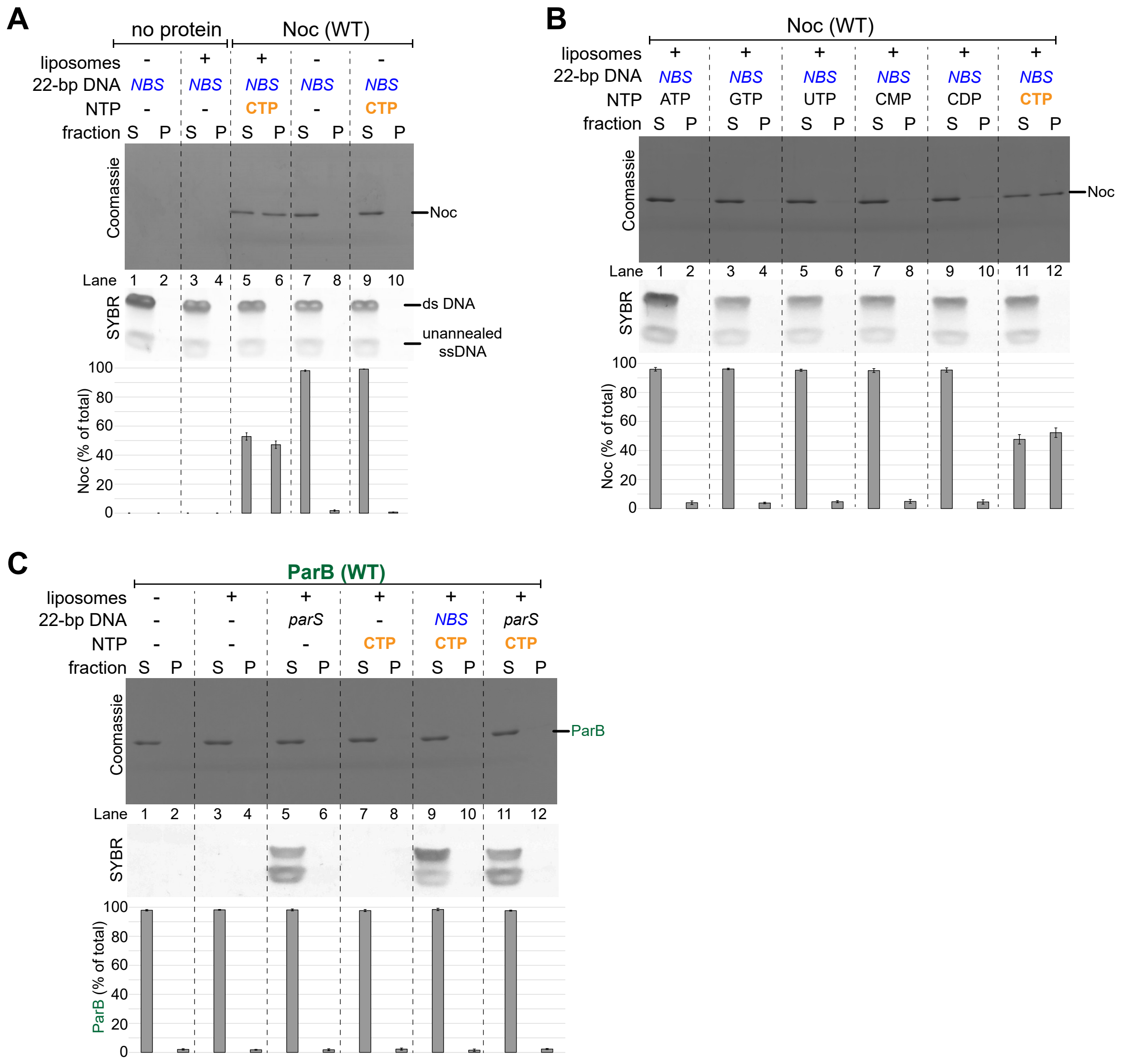

Figure S3. CTP and *NBS* DNA enable Noc binding to liposomes, related to Figure 3. (A) Analysis of *B. subtilis* Noc binding to membranes by a liposome co-sedimentation assay. A premix of 1.0 µM 22-bp linear *NBS* DNA ± 0.75 µM *B. subtilis* Noc protein ± 1.0 mM CTP ± 1.0 mg/mL liposomes was incubated at 22°C before ultracentrifugation. The resulting supernatant (S) and pellet (P) fractions were analyzed by SDS-PAGE. Without Noc, 22-bp *NBS* DNA did not co-sediment on its own (lanes 1-2) or with liposomes (lanes 3-4). Samples were also loaded onto a 20% TBE PAGE, and the gel was subsequently stained with Sybr Green for DNA. Quantification of Noc in each fraction is shown below each representative image. Error bars represent SEM from three replicates. **(B)** CTP but no other nucleotide enables Noc to co-sediment with liposomes. A premix of 0.75 µM *B. subtilis* Noc protein + 1.0 µM 22-bp *NBS* DNA ± 1.0 mM NTP + 1.0 mg/mL liposomes was incubated at 22°C for 5 min before ultracentrifugation. The resulting supernatant (S) and pellet (P) fractions were analyzed by SDS-PAGE. **(C)** *Caulobacter crescentus* ParB does not co-sediment with liposomes in any tested conditions. A premix of 0.75 µM *C. crescentus* ParB protein ± 1.0 µM 22-bp linear *parS*/*NBS* DNA ± 1.0 mM CTP ± 1.0 mg/mL liposomes was incubated at 22°C for 5 min before ultracentrifugation. The resulting supernatant (S) and pellet (P) fractions were analyzed by SDS-PAGE.

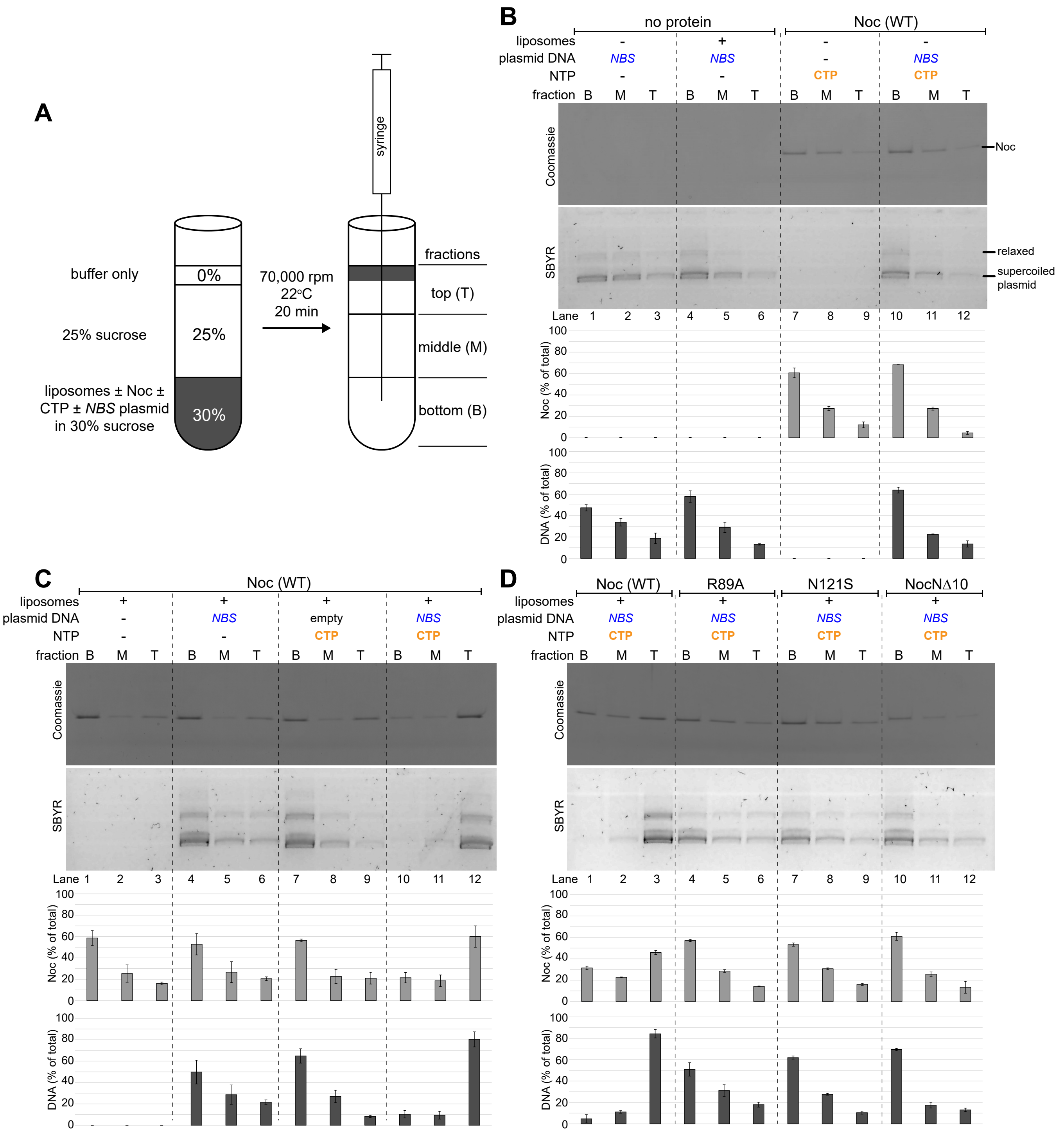

Figure legend (next page)

Figure S4. Liposome flotation assays show Noc can recruit *NBS* plasmid to the membrane in the presence of CTP, related to Figure 4. (A) The principle of a liposome flotation assay. Liposomes ± purified Noc ± CTP ± *NBS* plasmid were incubated in a 30% sucrose binding buffer. Buffer with 25% sucrose and 0% sucrose were subsequently layered on top sequentially. After ultracentrifugation, liposomes and associated protein/DNA migrate along the sucrose gradient i.e. floating to the uppermost fractions. Three equal fractions (bottom, middle, and top) were drawn out sequentially using a Hamilton syringe, and their protein and DNA contents were analyzed. **(B)** Control experiments: liposome flotation assays in which one component, either Noc protein, a 5-kb plasmid DNA, CTP, or liposomes, was omitted. **(C)** Analysis of *B. subtilis* Noc binding to membranes and the recruitment of plasmid DNA to membranes by a liposome flotation assay. A premix of 0.75 µM *B. subtilis* Noc ± 100 nM 5-kb plasmid DNA ± 1.0 mM CTP ± 1.0 mg/mL liposomes was incubated at 22°C for 5 min before ultracentrifugation. Either an empty plasmid or an *NBS*-harboring plasmid was employed in this assay. The resulting fractions (Bottom B, Middle M, and Top T) were analyzed by SDS-PAGE. Samples were also loaded onto a 1% agarose gel and was subsequently stained with Sybr Green for DNA. Quantification of Noc or DNA in each fraction is shown below each representative image. Error bars represent SEM from three replicates. **(D)** Other Noc variants, Noc (R89A), Noc (N121S), and NocNΔ10, were also analyzed in a liposome flotation assay. A premix of 0.75 µM *B. subtilis* Noc protein (WT or mutants) + 100 nM *NBS* plasmid + 1.0 mM CTP + 1.0 mg/mL liposomes was ultracentrifuged, and the resulting fractions were analyzed for protein and DNA contents.

A

```
B. subtilis Noc            MKHSFSRFFGLGEKEQEPEIAEHDTNKEEILEIPVNAIVPNRFQPRTIFSDEKIKELAMT  60
G. thermoleovorans Noc     MKHPFSRLFSFGEKEQEEM--EEKQEREEVRHIPVKSIIPNRFQPRTMFDEEKIDELALT  58
                           *** ***:*.:******    *.. ::**: .***::*:********:*.:***.***:*
                              α1            α2     α2-β1 linker

B. subtilis Noc            IHTHGIIQPIVVRHTEEEGQYELIAGERRWRAVQSLEWEKIPAIIKDFSDTETASVALIE  120
G. thermoleovorans Noc     IRTHGIIQPIVVREC-GNGRFEIIAGERRWRAVQKLGWTEIPAIIKNLNDKETASVALIE  117
                           *:***********.   :*::*:***********.* * :******::.*.*********
                                                      arginine-rich patch

B. subtilis Noc            NLQREELSSIEEAHAYARLLELHDLTQEALAQRLGKGQSTIANKLRLLKLPQPVQEAIME  180
G. thermoleovorans Noc     NLQREELTPIEEAMAYAKLIELHDLTQEALAQRLGKGQSTIANKLRLLKLPQEVQEALLQ  177
                           *******: **** ***:*:******************************** ****:::

B. subtilis Noc            KKITERHARALIPLKQPELQVTLLTEIIEKSLNVKQTEDRVVKMLEQGQRKPKPRRKAFS  240
G. thermoleovorans Noc     RAITERHARALIALKDKEKQLKLLQEIIDKQLNVKQTEDRVLKLLEAGERKPKPKRKAFS  237
                           : ********** **: * *:.** ***:*.**********:*:** *:*****:*****

B. subtilis Noc            RDTRIAMNTIRQSLSMVEDSGVKLNTEEEEFEEYIQLTIRIPK-    283   Identities: 72%
G. thermoleovorans Noc     RDMRIAVNTIRQSLSMVESSGVSVQSEEEEFDDYYQITIRIAKK    281
                           ** ***:***********.***.:::*****::* *:**** *
```

B

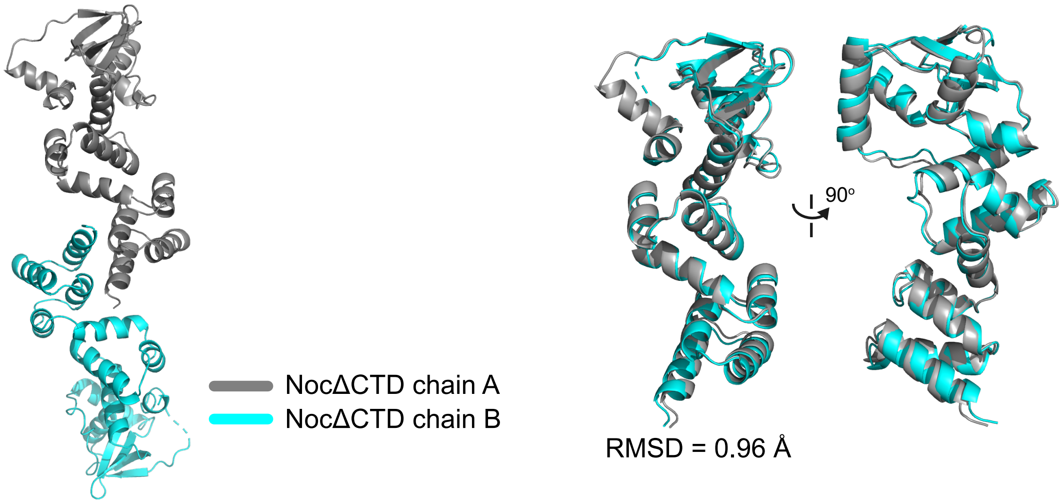

Figure S5. The composition of the asymmetric unit of *G. thermoleovorans* NocΔCTD crystal structure, related to Figure 5. (A) A sequence alignment between *B. subtilis* Noc and its homolog *G. thermoleovorans* Noc. The conserved arginine-rich patch that mediates CTP binding in *B. subtilis* ParB (Soh et al., 2019) is shown in the solid box. The sequences of helix α1, helix α2, and the α2-β1 connecting loop are also indicated on the sequence alignment. **(B)** The asymmetric unit of *G. thermoleovorans* NocΔCTD contains two copies of NocΔCTD monomers (left panel). Chains A and B are structurally similar, RMSD = 0.96 Å (right panel).

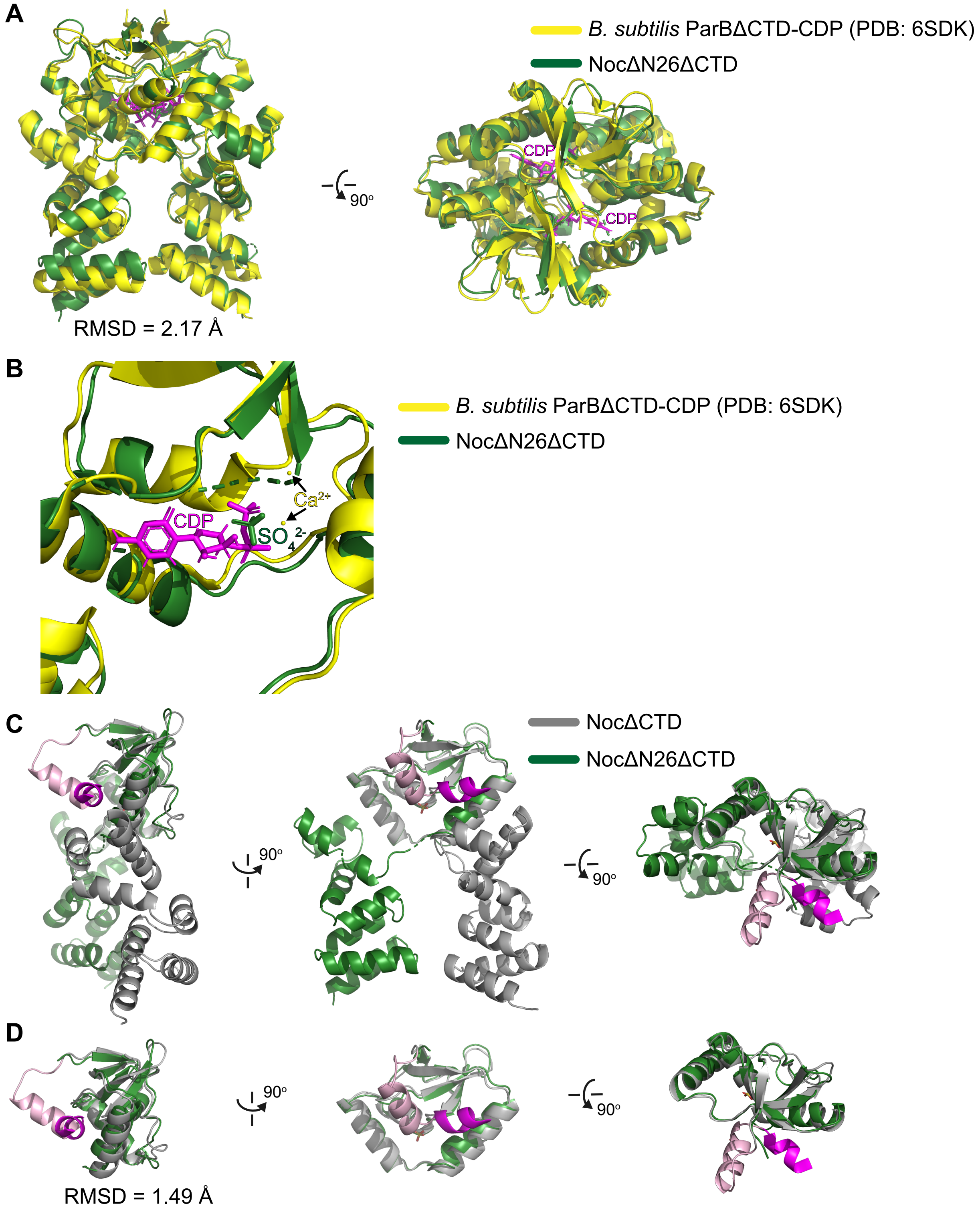

Figure S6. The conformation of *G. thermoleovorans* NocNΔ26ΔCTD is similar to that of a nucleotide-bound *B. subtilis* ParBΔCTD, related to Figure 6. (A) Superimposition between a *G. thermoleovorans* NocNΔ26ΔCTD dimer (green) and a *B. subtilis* ParBΔCTD-CDP dimer (yellow, PDB: 6SDK). CDP molecules are shown in magenta. **(B)** Magnification of the nucleotide-binding pocket of *B. subtilis* ParBΔCTD and *G. thermoleovorans* NocNΔ26ΔCTD. CDP and Ca^{2+} cations that belong to *B. subtilis* ParBΔCTD-CDP co-crystal structure are highlighted in magenta and yellow, respectively. In the *G. thermoleovorans* NocNΔ26ΔCTD structure, a sulfate ion (dark green) occupies a similar position to the β-phosphate group of CDP. **(C)** A superimposition at the N-terminal domains of a NocΔCTD monomer (grey) and a NocNΔ26ΔCTD monomer (green). The amphipathic helix α1 and helix α2 are shown in magenta and pink, respectively. **(D)** Same as panel B, but only the N-terminal domain (NTD) is shown for clarity.

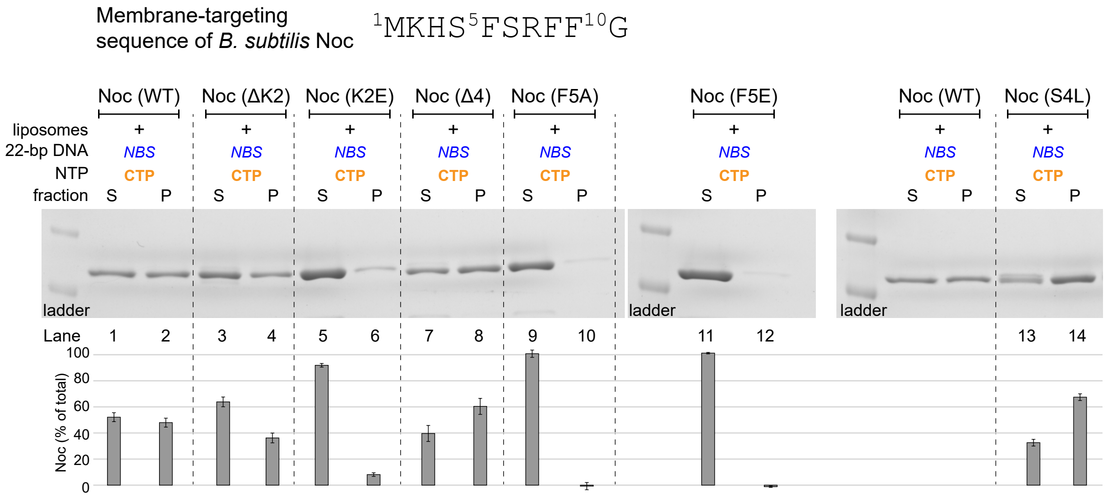

Figure S7. Effects of N-terminal substitutions and deletions on *B. subtilis* Noc-liposomes interaction, related to Figure 6. A premix of 1.0 μM 22-bp linear *NBS* DNA + 1.0 μM *B. subtilis* Noc protein (WT/mutants) + 1.0 mM CTP + 1.0 mg/mL liposomes was incubated at 22ºC before ultracentrifugation. The resulting supernatant (S) and pellet (P) fractions were analyzed by SDS-PAGE. Quantification of Noc in each fraction is shown below each representative image. Error bars represent SEM from three replicates.

TABLE S1. Bacterial strains. Related to STAR Methods.

Strains	Description	Source
E. coli Rosetta (DE3)	F^- *ompT hsdS*$_B$(r_B^- m_B^-) *gal dcm* (DE3) pRARE (chloramphenicol^R)	Merck
DWA117	*B. subtilis* 168CA (*trpC2*) *Δnoc::tet*	(Adams et al., 2015)
DWA206	*B. subtilis* 168CA (*trpC2*) *Δnoc::tet ΩamyE::[spc Pxyl-noc (WT)-myfp]*	(Adams et al., 2015)
DWA382	*B. subtilis* 168CA (*trpC2*) *Δnoc::tet ΩamyE::[spc Pxyl-nocNΔ10-myfp]*	(Adams et al., 2015)
DWA546	*B. subtilis* 168CA (*trpC2*) *Δnoc::tet ΩamyE::[spc Pxyl-noc (R89A)-myfp]*	(Adams et al., 2015)
4746	*B. subtilis* 168CA (*trpC2*) *Δnoc::tet ΩamyE::[spc Pxyl-noc (N121S)-yfpmut1]*	This study
DWA564	*B. subtilis* 168CA (*trpC2*) *Δnoc::tet ΔminCD::kan ΩamyE::[spc Pxyl-noc (WT)-myfp]*	(Adams et al., 2015)
DWA566	*B. subtilis* 168CA (*trpC2*) *Δnoc::tet ΔminCD::kan ΩamyE::[spc Pxyl-nocNΔ10-myfp]*	(Adams et al., 2015)
DWA600	*B. subtilis* 168CA (*trpC2*) *Δnoc::tet ΔminCD::kan ΩamyE::[spc Pxyl-noc (R89A)-myfp]*	(Adams et al., 2015)
4747	*B. subtilis* 168CA (*trpC2*) *Δnoc::tet ΔminCD::kan ΩamyE::[spc Pxyl-noc (N121S)-yfpmut1]*	This study

TABLE S2. DNA, plasmids, and oligos. Related to STAR Methods.

Plasmids	Description	Source
pET21b::*Bacillus subtilis noc-his*$_6$	Overexpression of a C-terminally His$_6$-tagged *B. subtilis* Noc, carbenicillin^R	This study
pET21b::*Bacillus subtilis nocNΔ10-his*$_6$	Overexpression of a C-terminally His$_6$-tagged *B. subtilis* Noc that lacks the first 10 amino acids, carbenicillin^R	This study
pET21b::*Bacillus subtilis noc (R89A)-his*$_6$	Overexpression of a C-terminally His$_6$-tagged *B. subtilis* Noc (R89A), carbenicillin^R	This study
pET21b::*Bacillus subtilis noc (N121S)-his*$_6$	Overexpression of a C-terminally His$_6$-tagged *B. subtilis* Noc (N121S), carbenicillin^R	This study
pET21b::*Bacillus subtilis noc (E29C)-his*$_6$	Overexpression of a C-terminally His$_6$-tagged *B. subtilis* Noc (E29C), carbenicillin^R	This study
pET21b::*Bacillus subtilis noc (E29C R89A)-his*$_6$	Overexpression of a C-terminally His$_6$-tagged *B. subtilis* Noc (E29C R89A), carbenicillin^R	This study
pET21b::*Bacillus subtilis noc (E29C N121S)-his*$_6$	Overexpression of a C-terminally His$_6$-tagged *B. subtilis* Noc (E29C N121S), carbenicillin^R	This study
pET21b::*Bacillus subtilis noc ΔK2-his*$_6$	Overexpression of a C-terminally His$_6$-tagged *B. subtilis* Noc that lacks the lysine at position 2, carbenicillin^R	This study
pET21b::*Bacillus subtilis noc Δ4-his*$_6$	Overexpression of a C-terminally His$_6$-tagged *B. subtilis* Noc that lacks the first 4 amino acids, carbenicillin^R	This study
pET21b::*Bacillus subtilis noc (K2E)-his*$_6$	Overexpression of a C-terminally His$_6$-tagged *B. subtilis* Noc (K2E), carbenicillin^R	This study
pET21b::*Bacillus subtilis noc (S4L)-his*$_6$	Overexpression of a C-terminally His$_6$-tagged *B. subtilis* Noc (S4L), carbenicillin^R	This study
pET21b::*Bacillus subtilis noc (F5A)-his*$_6$	Overexpression of a C-terminally His$_6$-tagged *B. subtilis* Noc (F5A), carbenicillin^R	This study
pET21b::*Bacillus subtilis noc (F5E)-his*$_6$	Overexpression of a C-terminally His$_6$-tagged *B. subtilis* Noc (F5E), carbenicillin^R	This study
pET21b::*Bacillus subtilis nocNΔ10 (E29C)-his*$_6$	Overexpression of a C-terminally His$_6$-tagged *B. subtilis* Noc (E29C) variant that also lacks the first 10 amino acids, carbenicillin^R	This study
pET21b::*Geobacillus thermoleovorans NocΔCTD*	Overexpression of a C-terminally His$_6$-tagged *G. thermoleovorans* Noc that lacks the last 42 amino acids, carbenicillin^R	This study
pET21b::*Geobacillus thermoleovorans NocNΔ26ΔCTD*	Overexpression of a C-terminally His$_6$-tagged *G. thermoleovorans* Noc that lacks the first 26 amino acids and the last 42 amino acids, carbenicillin^R	This study
pMCS5-4x*NBS*	pMCS5 plasmid that harbors four *NBS* sites, tetracycline^R	This study

pMCS5::empty	pMCS5 plasmid with an intact multiple cloning site, tetracycline^R	(Thanbichler et al., 2007)
pET21b::*Caulobacter crescentus parB-his$_6$*	Overexpression of a C-terminally His$_6$-tagged *C. crescentus* ParB, carbenicillin^R	Gift from Christine Jacobs-Wagner (Lim et al., 2014)
pSG4926	*bla amyE' spc Pxyl-noc-yfpmut1 'amyE*; shuttle plasmid harboring a xylose-inducible *noc-yfp* fusion for integration at the *B. subtilis amyE* locus	(Wu et al., 2009)
pSG4926 N121S	*bla amyE' spc Pxyl-noc (N121S)-yfpmut1 'amyE*; *B. subtilis* plasmid harboring a xylose-inducible *noc (N121S)-yfp* fusion for integration at the *B. subtilis amyE* locus	This study
**Oligos/gBlocks**		
170bp-*parS*	CGCCAGGGTTTTCCCAGTCACGACGTTGTAAAACGACGGCCAGAATTCGCAACGTGTGTTTCACGTGAAACAGCCTTGAACTGATAACGACTCTATCATTGATAGAGTGTTCTCTCCACG**GGATCC**CCAGGCATGCAAGCTTGGCGTAATCATGGTCATAGCTGTTTCCT	(Jalal et al., 2020a)
170bp-*NBS*	CGCCAGGGTTTTCCCAGTCACGACGTTGTAAAACGACGGCCAGAATTCGCAACGTGTATTTCCCGGGAAATAGCCTTGAACTGATAACGACTCTATCATTGATAGAGTGTTCTCTCCACG**GGATCC**CCAGGCATGCAAGCTTGGCGTAATCATGGTCATAGCTGTTTCCT	This study
22bp-*parS*	GGATGTTTCACGTGAAACATCC	(Jalal et al., 2020b)
22bp-*NBS*	GGATATTTCCCGGGAAATATCC	This study
260bp-4x*NBS* gBlocks	GCCCAGGCCCTGGAGCGCATCTCCGGCTATTTCCCGGGAAATAACGTTCTTGGACGGGTCTTTTCGACCTTCTGTATCGGCAAGTGATATTTCCCGGGAAATATCCCCAATATTGTCCACAGGCCGCTCACAGCTGCGGTGGGGTATTTCCCGGGAAATACCGATGAGTCACATCGACCCGCTCGCCTGATTGGCGTATAGATCTATTTCCCGGGAAATACTCGAACCCAGGGGCGTTCGCATTGAGGCGAGCGTCTTG	This study
AJ65	TAACTTTAAGAAGGAGATATACATATGTTGGGTGAAAAGGAGCAAGAACCG	This study
AJ66	GGTGGTGCTCGAGTGCGGCCGCAAGCTTTTTTGGAATACGGATTGTAAGCTG	This study
AJ73	CTGAACCGCGCGCCACGCGCGTTCTCCCGCAAT	This study
AJ74	ATTGCGGGAGAACGCGCGTGGCGCGCGGTTCAG	This study
AJ76	GGTGGTGCTCGAGTGCGGCCGCAAGCTTGTCGCGGGAAAAGGCTTTACGTTTAGG	This study
AJ81	TAACTTTAAGAAGGAGATATACATATGGAAGAGGTCCGTCACATCCCCGTCAAA	This study
AJ84	GATACCAATAAGGAATGCATTTTAGAAATTCCA	This study
AJ85	TGGAATTTCTAAAATGCATTCCTTATTGGTATC	This study
AJ86	GTGGCCTTAATTGAGTCTTTGCAACGCGAGGAG	This study
AJ87	CTCCTCGCGTTGCAAAGACTCAATTAAGGCCAC	This study
M13-F	CGCCAGGGTTTTCCCAGTCACGAC	Lab stock
M13-R	ATGGTCATAGCTGTTTCCT	Lab stock
Noc(N121S)-F	GTGGCGTTAATCGAAAGCTTGCAGCGGGAAGAA	This study
Noc(N121S)-R	TTCTTCCCGCTGCAAGCTTTCGATTAACGCCAC	This study

The sequences of *parS/NBS* are underlined. The sequence of BamHI recognition site is bold.

TABLE S3. X-ray data collection and processing statistics. Related to Figures 5 and 6.

**Structure**	***G. thermoleovorans*** **NocΔCTD - iodide**	***G. thermoleovorans*** **NocΔCTD - native**	***G. thermoleovorans*** **NocNΔ26ΔCTD**
Data collection			
Diamond Light Source beamline	I04	I04-1	I04
Wavelength (Å)	1.800	0.912	0.980
Detector	Eiger2 XE 16M	Pilatus 6M-F	Eiger2 XE 16M
Resolution range (Å)	96.61 – 3.40 (3.67 – 3.40)	84.89 – 2.50 (2.60 – 2.50)	37.44 – 2.95 (3.13 – 2.95)
Space Group	$P2_13$	$P2_13$	$C222_1$
Cell parameters (Å/°)	$a = b = c = 136.6$	$a = b = c = 146.8$	$a = 105.1$, $b = 106.6$, $c = 42.2$
Total no. of measured intensities	2147484 (426036)	1458851 (163435)	66966 (10641)
Unique reflections	11990 (2445)	36704 (4119)	5285 (835)
Multiplicity	179.1 (174.2)	39.7 (39.7)	12.6 (12.0)
Mean $I/\sigma(I)$	15.9 (3.3)	28.5 (1.8)	5.5 (1.5)
Completeness (%)	100.0 (100.0)	100.0 (100.0)	100.0 (100.0)
R_{merge}[a]	0.500 (3.392)	0.091 (2.654)	0.281 (1.343)
R_{meas}[b]	0.501 (3.402)	0.093 (2.688)	0.293 (1.399)
$CC_{½}$[c]	0.999 (0.894)	1.000 (0.670)	0.997 (0.885)
Wilson B value (Å^2)	88.7	68.0	38.2
Refinement			
Resolution range (Å)	-	84.89 – 2.50 (2.57 – 2.50)	37.44 – 2.95 (3.03 – 2.95)
Reflections: working/free[d]	-	34843/1794	4752/522
R_{work}[e]	-	0.210 (0.328)	0.267 (0.438)
R_{free}[e]	-	0.240 (0.386)	0.288 (0.443)
Ramachandran plot: favored/allowed/disallowed[f] (%)	-	98.1/1.9/0.0	98.0/2.0/0.0
R.m.s. bond distance deviation (Å)	-	0.010	0.007
R.m.s. bond angle deviation (°)	-	1.55	1.33
Mean B factors: protein/sulfate/water/ overall (Å^2)	-	85/118/71/86	74/70/0/74
**PDB accession code**		**7NFU**	**7NG0**

Values in parentheses are for the outer resolution shell.
[a] $R_{merge} = \sum_{hkl} \sum_i |I_i(hkl) - \langle I(hkl)\rangle| / \sum_{hkl} \sum_i I_i(hkl)$.
[b] $R_{meas} = \sum_{hkl} [N/(N-1)]^{1/2} \times \sum_i |I_i(hkl) - \langle I(hkl)\rangle| / \sum_{hkl} \sum_i I_i(hkl)$, where $I_i(hkl)$ is the ith observation of reflection *hkl*, $\langle I(hkl)\rangle$ is the weighted average intensity for all observations i of reflection *hkl* and N is the number of observations of reflection *hkl*.
[c] $CC_{½}$ is the correlation coefficient between symmetry equivalent intensities from random halves of the dataset.
[d] The dataset was split into "working" and "free" sets consisting of 95 and 5% of the data respectively. The free set was not used for refinement.
[e] The R-factors R_{work} and R_{free} are calculated as follows: $R = \sum(|F_{obs} - F_{calc}|)/\sum|F_{obs}|$, where F_{obs} and F_{calc} are the observed and calculated structure factor amplitudes, respectively.
[f] As calculated using MolProbity (Williams et al., 2018)
